# Comparative Analysis of Codon Usage Bias Patterns in Microsporidian Genomes

**DOI:** 10.1371/journal.pone.0129223

**Published:** 2015-06-09

**Authors:** Heng Xiang, Ruizhi Zhang, Robert R. Butler, Tie Liu, Li Zhang, Jean-François Pombert, Zeyang Zhou

**Affiliations:** 1 College of Animal Science and Technology, Southwest University, Chongqing 400715, China; 2 State Key Laboratory of Silkworm Genome Biology, Southwest University, Chongqing 400715, China; 3 Department of Biological and Chemical Sciences, Illinois Institute of Technology, Chicago, Illinois, United States of America; 4 Biotechnology Research Center, Southwest University, Chongqing 400715, China; 5 Department of Math and Information, China West Normal University, Nanchong, Sichuan 637000, China; Institute of Plant Physiology and Ecology, CHINA

## Abstract

The sub-3 Mbp genomes from microsporidian species of the *Encephalitozoon* genus are the smallest known among eukaryotes and paragons of genomic reduction and compaction in parasites. However, their diminutive stature is not characteristic of all Microsporidia, whose genome sizes vary by an order of magnitude. This large variability suggests that different evolutionary forces are applied on the group as a whole. In this study, we have compared the codon usage bias (CUB) between eight taxonomically distinct microsporidian genomes: *Encephalitozoon intestinalis*, *Encephalitozoon cuniculi*, *Spraguea lophii*, *Trachipleistophora hominis*, *Enterocytozoon bieneusi*, *Nematocida parisii*, *Nosema bombycis* and *Nosema ceranae*. While the CUB was found to be weak in all eight Microsporidia, nearly all (98%) of the optimal codons in *S*. *lophii*, *T*. *hominis*, *E*. *bieneusi*, *N*. *parisii*, *N*. *bombycis* and *N*. *ceranae* are fond of A/U in third position whereas most (64.6%) optimal codons in the *Encephalitozoon* species *E*. *intestinalis* and *E*. *cuniculi* are biased towards G/C. Although nucleotide composition biases are likely the main factor driving the CUB in Microsporidia according to correlation analyses, directed mutational pressure also likely affects the CUB as suggested by ENc-plots, correspondence and neutrality analyses. Overall, the *Encephalitozoon* genomes were found to be markedly different from the other microsporidians and, despite being the first sequenced representatives of this lineage, are uncharacteristic of the group as a whole. The disparities observed cannot be attributed solely to differences in host specificity and we hypothesize that other forces are at play in the lineage leading to *Encephalitozoon* species.

## Introduction

Microsporidia are spore-forming, single-celled fungal pathogens best known for their unique infection apparatus called the polar tube and for harboring species with the smallest reported nuclear genomes. Microsporidia as a group are highly diverse, with more than 1,500 distinct species infecting vertebrate and invertebrate hosts widely spread across the Tree of Life, and cause growing concerns due to their medical, environmental and economic relevance [1]. Their diversity is reflected at the genetic level, as the extreme levels of reduction encountered in the *Encephalitozoon* lineage [2–5] are not characteristic of the group. The microsporidian genetic paraphernalia vary by at least an order of magnitude, from as little as 2.3 Mbp [2] to more than 25 Mbp [6], with the underlying content and structure changing accordingly. These changes may reflect, at least in part, the different evolutionary pressures applied by the various host ranges with which each microsporidian species co-evolve, and a better understanding of these changes may lead to better predictions models about their zoonotic and lethal potentials.

The genetic code plays a critical role in living cells, but not all species use its built-in redundancy in the same way. Codon usage biases (CUB) are widespread across the Tree of Life and are affected by nucleotide composition [7], translation processes [8], tRNA abundance [9], gene function [10] and length [11], protein structure [12] and hydrophobicity [13], environment temperature [14] and other factors. In particular, the balance between gene mutation and natural selection determines the CUB [15]. The genetic code itself is not universal and deviations from the standard code can have profound impacts on the translational apparatus of the corresponding organisms. Conversely, irreversible modifications to a species’ translational apparatus can force it to adapt its CUB accordingly. With genome reduction often comes simplification and forced specialization at the expense of versatility.

Microsporidia have simpler ribosomes than their fungal relatives with sediment coefficients that are similar to that of prokaryotes [16]. This simplification could potentially limit the breadth of possible codon usage biases that they can adopt. While CUB in Microsporidia have been investigated to various degrees [4, 17–19], only one study addressed their CUB in a systematic, albeit succinct fashion [18]. Here, we expanded on previous studies by using a wider selection of eight taxonomically dispersed microsporidian representatives with available genomic sequences. By examining several statistics that characterize CUB, we identify several trends and uncover some implications for selection pressures affecting this idiomatic phylum.

## Materials and Methods

### Genomes and coding sequences

The annotated genomes of eight microsporidian species and their coding sequences (CDS) were obtained from GenBank (http://www.ncbi.nlm.nih.gov/genbank). The choice of the genomes investigated was based on the overall quality of their respective annotations, the diversity of hosts infected by the selected species, and the wide distribution of the selected species across the microsporidian phylogenetic tree (see Vossbrink and Debrunner-Vossbrink 2005 [20]). *Encephalitozoon intestinalis* ATCC 50506 [2], *Encephalitozoon cuniculi* GB-M1 [3], *Spraguea lophii* 42_110 [21], *Trachipleistophora hominis* [18], *Enterocytozoon bieneusi* H348 [22], *Nematocida parisii* ERTm1 [23], *Nosema bombycis* CQ1 [19] and *Nosema ceranae* BRL01 [24] featured a total of 1939, 1996, 2499, 3212, 3632, 2661, 4468 and 2060 annotated CDS, respectively. To minimize outliers caused by small sizes, only CDS of at least 300 bp were kept for downstream analyses. Thus, a total of 1770, 1960, 2461, 2476, 2932, 2464, 3740 and 2022 CDS for *E*. *intestinalis*, *E*. *cuniculi*, *S*. *lophii* and *T*. *hominis*, *E*. *bieneusi*, *N*. *parisii*, *N*. *bombycis* and *N*. *ceranae*, respectively, were analyzed.

### Nucleotide composition analyses

The GC content of the entire CDS (GC_cds_) as well as the first (P_1_), second (P_2_), and third (P_3_) codon position GC content were calculated using a custom PERL script (available on https://github.com/hxiang1019/calc_GC_content.git). To account for the inequality of α and γ at the third codon position [25], the three stop codons (UAA, UAG, and UGA) and the three codons for isoleucine (AUU, AUC, and AUA) were excluded in calculation of P_3_, and the two single codons for methionine (AUG) and tryptophan (UGG) were excluded from P_1_, P_2_, and P_3_. Neutrality plots were drawn using the average value of P_1_ and P_2_ (P_12_) as the vertical axis and the P_3_ as the horizontal axis. The nucleotide compositions of the third codon position (A_3_, U_3_, C_3_, and G_3_) were also obtained and used to calculate the AU-bias [A_3_/ (A_3_+U_3_)] and GC-bias [G_3_/ (G_3_+C_3_)]. The Parity rule 2 (PR2) plots were drawn based on AU-bias and GC-bias.

### Codon usage indices and ENc-plot

The Codon Adaptation Index (CAI), the Effective Number of Codons (ENc), and the third synonymous codon position GC content (GC_3s_) were calculated using CodonW (John Peden, http://www.molbiol.ox.ac.uk/cu, version 1.4.2) using *Saccharomyces cerevisiae* as reference [16]. The ENc vs GC_3s_ plots were generated from this data.

### RSCU and correspondence analyses

The relative synonymous codon usage (RSCU) was calculated using CodonW. The high- and low-expression gene datasets were defined as genes in the upper and lower 5% of CAI values for each microsporidian species. RSCU values of these two datasets were compared through a chi-squared test, and the codons whose usage frequency in the high-expression genes was significantly higher (*P*-value < 0.05) than in the low-expression genes were identified as the optimal codons [26]. Codons with RSCU values less than 0.1 were classified as rare codons. A heat map was drawn with CIMMiner (http://discover.nci.nih.gov/cimminer) [27] and clustered the microsporidian RSCU values using a Euclidean distance method and an Average Linkage cluster algorithm. The correspondence analysis (COA) [28] was performed with CodonW utilizing the RSCU values to compare the intra-genomic variation of 59 informative codons, partitioned along 59 orthogonal axes with 41 degrees of freedom. Correlation analyses, ANOVA and significance tests were performed with Microsoft Excel and SPSS 18.0 (http://www.spss.com/).

## Results

### Codon usage biases

Codon usage patterns for microsporidian genomes were investigated by calculating RSCU values (Table 1). The RSCU is the observed frequency of a codon divided by the expected one. If the RSCU is close to 1, synonymous codons are used without apparent biases. When the RSCU value is greater or less than 1, the codons investigated are used more or less frequently than expected, respectively.

**Table 1:** The RSCU analysis of the preferred codons (codons with RSCU > 1), the optimal codons and the rare codons for microsporidian genomes.

Amino acid	Codon	RCSU							
		*E*. *intestinalis*	*E*. *cuniculi*	*S*. *lophii*	*T*. *hominis*	*Ent*. *bieneusi*	*Nem*. *parisii*	*N*. *bombycis*	*N*. *ceranae*
Phe	UUU	1.31*	1.26*	1.67*	1.53*	1.90*	1.53*	1.74*	1.86*
	UUC	0.69	0.74	0.33	0.47	0.10	0.47	0.26	0.14
Leu	UUA	0.22	0.20	4.02*	2.04*	4.33*	3.21*	3.70*	3.33*
	UUG	0.92	0.77*	0.40	1.35*	0.49	0.47	0.65	0.71
	CUU	1.96*	**1.67**	0.71	**1.07**	0.71*	0.80*	1.13*	**1.13**
	CUC	0.66	0.91	0.15	0.34	0.04-	0.12	0.20	0.08-
	CUA	0.53	0.32	0.63*	0.73*	0.40*	1.02*	0.30	0.62
	CUG	1.71*	2.14*	0.09-	0.47	0.03-	0.38	0.03-	0.13
Ile	AUU	1.27*	1.28*	0.84	1.26*	1.55*	1.10*	1.70*	**1.55**
	AUC	0.86	0.73	0.16	0.45	0.09-	0.16	0.24	0.15
	AUA	0.88	0.99*	2.01*	1.29*	1.36*	1.74*	1.06*	1.31*
Met	AUG	1.00	1.00	1.00	1.00	1.00	1.00	1.00	1.00
Val	GUU	**1.26**	1.28*	**1.07**	1.49*	1.85*	1.03*	1.56*	**1.53**
	GUC	0.45	0.56	0.04-	0.39	0.11	0.18	0.24	0.20
	GUA	0.50	0.37	2.54*	1.28*	1.90*	2.09*	1.88*	1.96*
	GUG	1.78*	1.79*	0.35	0.83	0.15	0.70	0.32	0.31
Tyr	UAU	**1.01**	0.98*	1.82*	1.48*	1.87*	1.58*	1.55*	1.54*
	UAC	0.99*	**1.02**	0.18	0.52	0.13	0.42	0.45	0.46
Stop	UAA	**1.16**	0.00-	**2.32**	**1.57**	**2.42**	**2.16**	**1.75**	**2.02**
	UAG	0.41	0.00-	0.32	0.54	0.32	0.57	0.43	0.50
	UGA	**1.44**	0.00-	0.37	0.90	0.25	0.27	0.81	0.48
His	CAU	**1.13**	0.96*	1.80*	1.53*	1.84*	1.53*	1.68*	1.61*
	CAC	0.87*	**1.04**	0.20	0.47	0.16	0.47	0.32	0.39
Gln	CAA	0.59	0.36	**1.50**	1.47*	1.87*	1.23*	1.86*	1.53*
	CAG	1.41*	1.64*	0.50	0.53	0.13	0.77	0.14	0.47
Asn	AAU	0.83	0.95*	1.67*	1.43*	1.85*	1.68*	1.79*	1.73*
	AAC	1.17*	**1.05**	0.33	0.57	0.15	0.32	0.21	0.27
Lys	AAA	0.37	0.29	1.55*	1.49*	1.85*	1.44*	1.62*	1.67*
	AAG	1.63*	1.71*	0.45	0.51	0.15	0.56	0.38	0.33
Asp	GAU	1.00	0.98*	1.91*	1.59*	1.85*	1.60*	1.88*	**1.71@**
	GAC	1.00*	**1.02**	0.09-	0.41	0.15	0.40	0.12	0.29
Glu	GAA	0.64	0.45	**1.67**	1.53*	1.87*	1.53*	1.67*	1.70*
	GAG	1.36*	1.55*	0.33	0.47	0.13	0.47	0.33	0.30
Ser	UCU	**1.27**	**1.30**	**1.49**	1.18*	1.65*	1.37*	2.22*	**1.93**
	UCC	0.51	0.46	0.14	0.42	0.20	0.30	0.46	0.21
	UCA	0.46	0.28	0.82	1.60*	2.23*	1.39*	1.23*	**1.38**
	UCG	1.23*	1.09*	0.09-	0.65	0.12	0.18	0.13	0.26
	AGU	1.04*	1.27*	3.15*	1.41*	1.58*	2.31*	1.90*	1.82*
	AGC	1.49*	1.60*	0.32	0.74	0.21	0.44	0.06-	0.40
Pro	CCU	**1.24**	1.28*	2.15*	1.22*	1.56*	1.20*	2.12*	1.93*
	CCC	0.67	0.74	0.31	0.37	0.13	0.38	0.39	0.28
	CCA	0.99	0.65	**1.43**	1.85*	2.20*	2.03*	1.38*	**1.54**
	CCG	1.11*	1.33*	0.10	0.55	0.10	0.38	0.11	0.25
Thr	ACU	0.63	0.84	1.89*	1.03*	1.69*	1.39*	2.03*	**1.35**
	ACC	0.54	0.63	0.56	0.53	0.18	0.32	0.33	0.26
	ACA	**1.75**	0.91	**1.44**	1.81*	2.00*	2.00*	1.39*	2.19*
	ACG	1.08*	1.61*	0.11	0.63	0.13	0.29	0.24	0.20
Ala	GCU	0.58	0.75	1.81*	1.27*	1.74*	1.07*	2.24*	**1.31**
	GCC	0.69	0.66	0.18	0.48	0.15	0.41	0.43	0.27
	GCA	2.09*	**1.48**	**1.80**	1.71*	2.02*	2.25*	1.29*	2.17*
	GCG	0.64*	1.11*	0.22	0.54	0.09-	0.27	0.04-	0.26
Cys	UGU	0.99	0.93*	1.87*	1.41*	1.72*	1.51*	1.91*	**1.61**
	UGC	**1.01**	**1.07**	0.13	0.59	0.28	0.49	0.09-	0.39
Trp	UGG	1.00	1.00	1.00	1.00	1.00	1.00	1.00	1.00
Arg	CGU	0.18	0.23	0.40	0.85	0.77	0.53*	0.27	0.39
	CGC	0.14	0.19	0.03-	0.34	0.08-	0.16	0.00-	0.21
	CGA	0.67*	0.22	0.11	1.09*	1.42*	0.17	0.25	0.46
	CGG	0.81*	1.08*	0.02-	0.33	0.11	0.22	0.05-	0.06-
	AGA	**1.95**	**1.53**	4.97*	2.51*	3.17*	3.72*	3.45*	4.22*
	AGG	2.25*	2.76*	0.46	0.87	0.46	**1.21**	1.97*	0.66
Gly	GGU	0.17	0.34	2.66*	1.60*	1.19*	1.37*	1.13*	**1.27**
	GGC	0.23	0.65	0.11	0.52	0.12	0.59	0.06-	0.34
	GGA	**2.01**	**1.27**	**1.09**	1.53*	2.53*	**1.32**	1.81*	2.12*
	GGG	1.59*	1.74*	0.14	0.36	0.16	0.73	1.00*	0.27

Both the sign * (*P*-value < 0.01) and @ (0.01 < *P*-value < 0.05) represent the optimal codons, while the sign - (RSCU < 0.10) denotes the rarely used codons. The preferred codons (RSCU > 1) are in bold.

**Fig 1:**
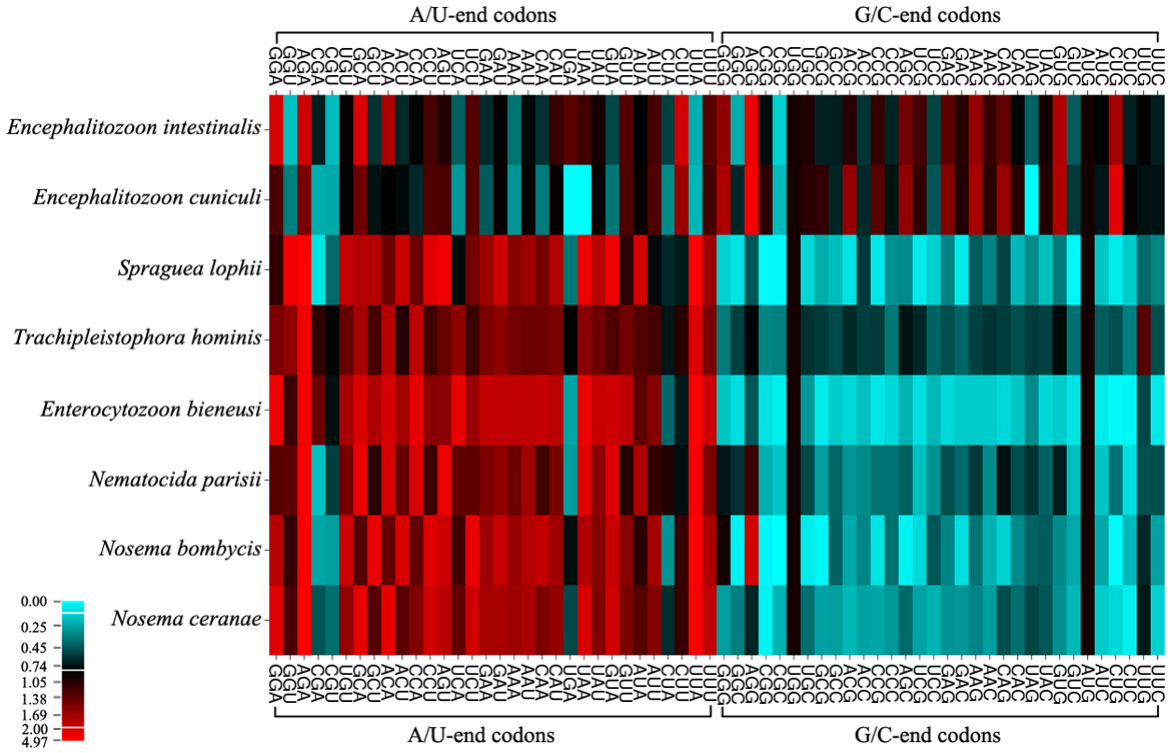
Heat map of RSCU values in microsporidian genomes. The heat-map was drawn with CIMminer, using the quantile binning method. Bigger RSCU values, suggesting more frequent codon usage, are represented with darker shades of red. Six Microsporidia (*S*. *lophii*, *T*. *hominis*, *E*. *bieneusi*, *N*. *parisii*, *N*. *bombycis*, *N*. *ceranae*) strongly prefer the A/U-end codons, while *Encephalitozoon* genus (*E*. *intestinalis*, *E*. *cuniculi*) displays a more varied distribution.

The preferred codons (RSCU > 1, Table 1; in bold) in *S*. *lophii*, *T*. *hominis*, *E*. *bieneusi*, *N*. *parisii*, *N*. *bombycis*, and *N*. *ceranae* are strongly biased towards A/U bases in third position, in contrast to *E*. *intestinalis* and *E*. *cuniculi* where more than half of the codons end with G or C (Table 2). Optimal codons (shown in * or @, Table 1) identified by chi-squared tests are similarly biased. Nearly all of the optimal codons in *S*. *lophii* (17 A/U-end in 17 optimal codons), *T*. *hominis* (28 A/U-end in 29 optimal codons), *E*. *bieneusi* (29 A/U-end in 29 optimal codons), *N*. *parisii* (28 A/U-end in 28 optimal codons), *N*. *bombycis* (27 A/U-end in 29 optimal codons), and *N*. *ceranae* (17 A/U-end in 17 optimal codons) are A/U-end whereas more than half of the optimal codons in *E*. *intestinalis* (17 G/C-end in 23 optimal codons) and *E*. *cuniculi* (14 G/C-end in 25 optimal codons) are G/C-end (Table 2). When clustering these biases according to a heat map (Fig 1), these values display a remarkable difference between the *Encephalitozoon* species and the other six microsporidians.

**Table 2:** The summary of the preferred codons, the optimal codons and the rare codons for microsporidian genomes.

Codon type	Codon 3^rd^ base	*E*. *intestinalis*	*E*. *cuniculi*	*S*. *lophii*	*T*. *hominis*	*Ent*. *bieneusi*	*Nem*. *parisii*	*N*. *bombycis*	*N*. *ceranae*
RSCU > 1	A/U/G/C	28	28	25	30	28	29	29	28
	A/U	15	10	25	29	28	28	28	28
	G/C	13	18	0	1	0	1	1	0
	A	6	3	12	14	14	14	13	13
	U	9	7	13	15	14	14	15	15
	G	10	12	0	1	0	1	1	0
	C	3	6	0	0	0	0	0	0
Optimal	A/U/G/C	23	25	17	29	29	28	29	17
	A/U	6	11	17	28	29	28	27	17
	G/C	17	14	0	1	0	0	2	0
	A	2	1	6	14	14	12	12	10
	U	4	10	11	14	15	16	15	7
	G	12	13	0	1	0	0	2	0
	C	5	1	0	0	0	0	0	0
Rare	A/U/G/C	0	3	6	0	5	0	7	2

### Correlation analyses

Nucleotide composition is an important factor influencing CUB, and the mean values of all of the microsporidian GC_cds_ are similar to their reported overall genomic GC content (Table 3). The GC_cds_ of *S*. *lophii* (25.62%), *T*. *hominis* (39.60%), *E*. *bieneusi* (32.07%), *N*. *parisii* (36.37%), *N*. *bombycis* (30.57%), and *N*. *ceranae* (27.23%) are low, while the GC_cds_ of *E*. *intestinalis* (42.01%) and *E*. *cuniculi* (47.59%) are notably higher. The correlation analysis (Table 4) shows that the GC_cds_, P_1_, P_2_ and P_3_ are significantly related to each other for all eight Microsporidia.

**Table 3:** Genome features (Genome size, GC_genome_, No. of predicted gene and CDS) obtained from the genome database of NCBI (http://www.ncbi.nlm.nih.gov/genome/), and GC contents calculated in this paper for microsporidia genomes.

Organism	*E*. *intestinalis*	*E*. *cuniculi*	*S*. *lophii*	*T*. *hominis*	*Ent*. *bieneusi*	*Nem*. *parisii*	*N*. *bombycis*	*N*. *ceranae*
Size (Mbp)	2.22	2.50	4.98	8.50	3.86	4.07	15.69	7.86
GCgenome	41.5%	47.3%	23.4%	34.1%	33.7%	34.4%	30.8%	25.3%
Genes	2,011	2,029	2,596	3,253	3,806	2,724	4,468	2,678
CDS	1,939	1,996	2,499	3,212	3,632	2,661	4,468	2,060
CDSused	1,770	1,960	2,461	2,476	2,932	2,464	3,740	2,022
GCcds	42.01%	47.59%	25.62%	39.60%	32.07%	36.37%	30.57%	27.23%
GC3s	44.23%	55.25%	15.50%	41.63%	30.69%	32.72%	23.69%	18.11%
P1	47.42%	51.03%	34.55%	45.48%	42.28%	40.36%	39.03%	36.28%
P2	34.37%	37.73%	25.74%	32.14%	30.53%	34.13%	28.79%	27.08%
P12	40.90%	44.38%	30.15%	38.81%	36.41%	37.25%	33.91%	31.68%
P3	45.82%	56.90%	16.49%	42.93%	31.64%	34.83%	24.51%	19.26%
ENc	50.41	50.49	37.04	51.91	44.96	47.37	42.29	40.86

**Table 4:** Correlation analysis among GC_cds_, P_1_, P_2_, P_12_, P_3_ and ENc for eight Microsporidia.

	GC_cds_	P_1_	P_2_	P_12_	P_3_
P1	0.583*/0.615* 0.816*/0.725* 0.896*/0.666* 0.717*/0.768*				
P2	0.515*/0.534* 0.793*/0.595* 0.812*/0.699* 0.747*/0.805*	0.102*/0.209* 0.476*/0.370* 0.674*/0.226* 0.377*/0.458*			
P12	0.747*/0.742* 0.946*/0.810* 0.940*/0.877* 0.889*/0.930*	0.694*/0.726* 0.872*/0.819* 0.932*/0.812* 0.821*/0.856*	0.745*/0.787* 0.827*/0.810* 0.888*/0.722* 0.813*/0.835*		
P3	0.671*/0.580* 0.507*/0.792* 0.939*/0.792* 0.543*/0.539*	0.160*/0.103* 0.190*/0.454* 0.767*/0.420* 0.130*/0.160*	-0.023/-0.120* 0.219*/0.239* 0.646*/0.340* 0.166*/0.263*	0.082*/-0.021 0.246*/0.420* 0.781*/0.481* 0.168*/0.254*	
ENc	0.016/-0.287* 0.446*/0.143* 0.611*/0.486* 0.234*/0.423*	-0.017/-0.068* 0.252*/0.095* 0.538*/0.265* 0.061*/0.191*	-0.102*/-0.033 0.230*/0.041* 0.352*/0.274* 0.010/0.209*	-0.080*/-0.061* 0.291*/0.079* 0.503*/0.338* 0.048*/0.240*	0.101*/-0.385* 0.627*/0.194* 0.643*/0.545* 0.471*/0.663*

The eight Spearman’s rank correlation coefficients (ρ) are the results of *E*. *intestinalis / E*. *cuniculi*, *S*. *lophii / T*. *hominis*, *Ent*. *bieneusi / Nem*. *parisii*, *N*. *bombycis / N*. *ceranae*, respectively. *P*-values < 0.01 are indicated by asteriskes (*).

In addition, correlations between ENc and GC_cds_, P_1_, P_2_ and P_3_ were also investigated. ENc relates the overall synonymous codon usage, ranging from only 20 codons being used for each of the 20 amino acids, to all 61 codons being used randomly [29]. In Table 4, the ENc is significantly correlated to GC_cds_, P_1_, P_2_ and P_3_ for Microsporidia. Correlations between ENc and GC_cds_, P_1_, P_2_ and P_3_ were either negative or null for *Encephalitozoon* species but positive for the other six Microsporidia.

**Fig 2:**
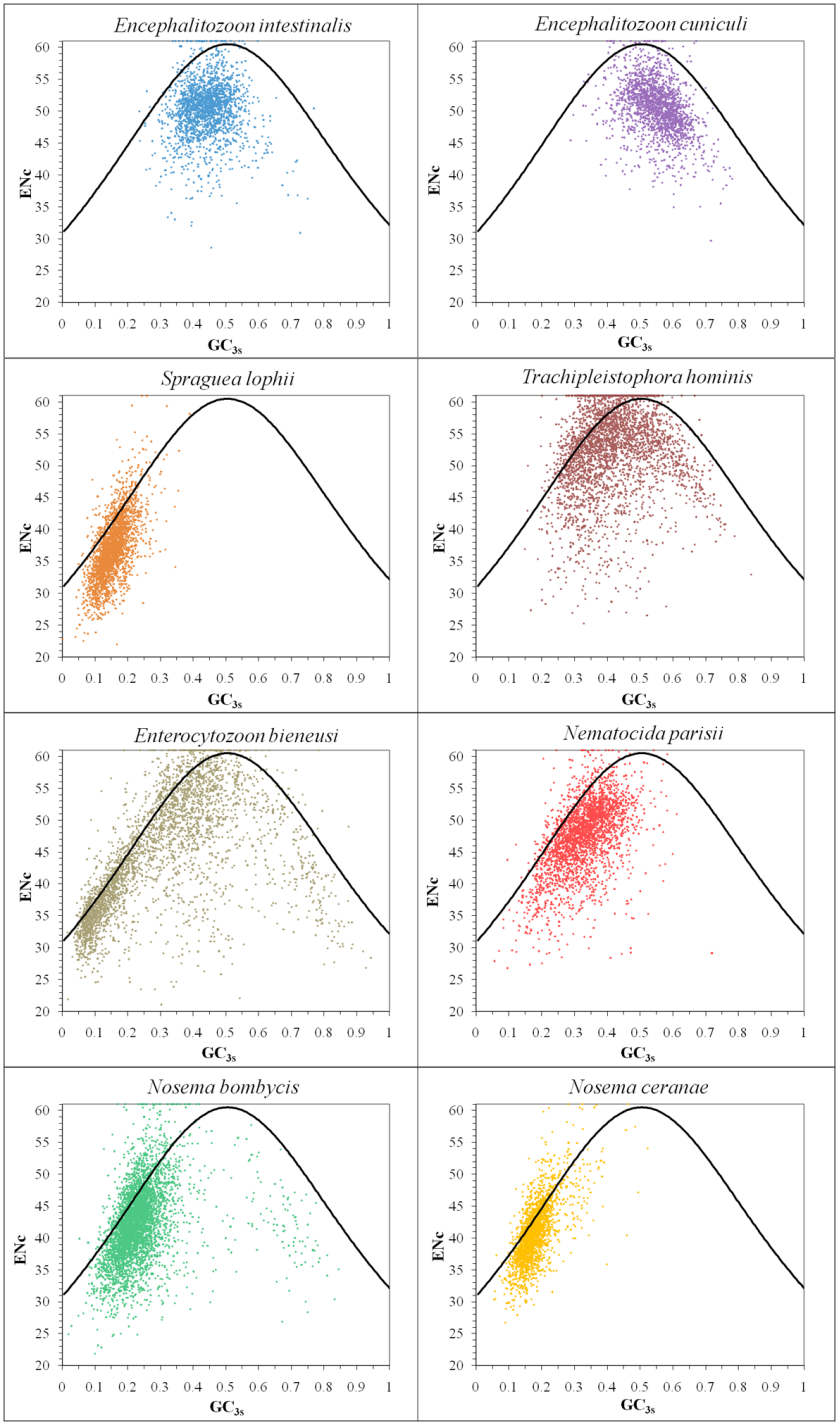
The ENc vs. GC_3s_ plots of microsporidian genomes. The standard curve ENc = 2+GC_3s_+29/[GC_3s_^2^+(1-GC_3s_)^2^] represents the expected ENc to GC_3s_. Most microsporidian genes are far away from the curve, showing that their codon usage pattern might be affected by other factors besides nucleotide composition. Some genes with the ENc score of 61 display no bias and use all the 61 sense codons.

To judge whether the nucleotide composition is the only factor to influence CUB for Microsporidia, the ENc-plot (Fig 2) was drawn. If genes follow the standard curve ENc = 2+GC_3s_+29/[GC_3s_^2^+(1-GC_3s_)^2^], the microsporidian CUB is determined primarily by the nucleotide composition [29]. In Fig 2, the distribution of most genes below the standard curve indicates that there are other factors acting on microsporidian CUB.

### Parity Rule 2 plot analyses

**Fig 3:**
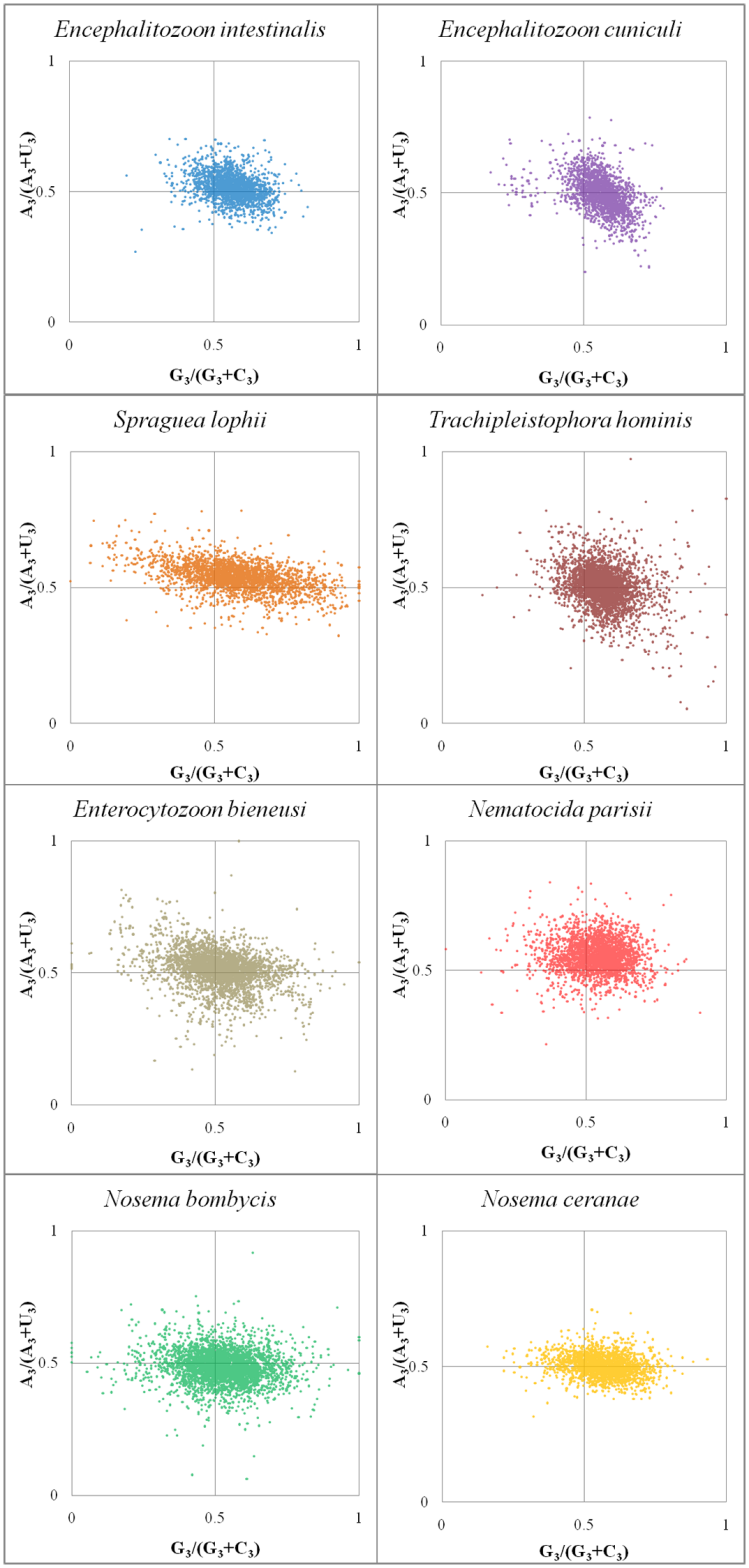
The PR2-bias plots of microsporidian genomes. Genes are plotted based on their GC bias [G_3_/(G_3_+C_3_)] and AU bias[A_3_/(A_3_+U_3_)] in the third codon position. The mean GC-biases of *E*. *intestinalis*, *E*. *cuniculi*, *S*. *lophii*, *T*. *hominis*, *E*. *bieneusi*, *N*. *parisii*, *N*. *bombycis*, and *N*. *ceranae* are 0.560, 0.558, 0.574, 0.569, 0.514, 0.538, 0.526, and 0.564, respectively; while their mean AU-biases are 0.517, 0.497, 0.538, 0.503, 0.514, 0.554, 0.485, and 0.502, respectively.

Examining the codons with RSCU > 1 and the optimal codons, species from the *Encephalitozoon* genus prefer G-end over C-end, despite the other microsporidians preferring A and U ends to a roughly equivalent degree (Table 2). Because of this bias of guanine over cytosine, all codons were examined by PR2 plot analysis (Fig 3). In the PR2 plot, the mean GC-biases [G_3_/(G_3_+C_3_)] of *E*. *intestinalis*, *E*. *cuniculi*, *S*. *lophii*, *T*. *hominis*, *E*. *bieneusi*, *N*. *parisii*, *N*. *bombycis*, and *N*. *ceranae* are 0.560, 0.558, 0.574, 0.569, 0.514, 0.538, 0.526, and 0.564; while their mean AU-biases [A_3_/(A_3_+U_3_)] are 0.517, 0.497, 0.538, 0.503, 0.514, 0.554, 0.485, and 0.502, respectively. In several of the species the PR2 plot highlights a slight preference for third position G over C. In a gene where CUB is only influenced by nucleotide composition, the third positions should have the identical distribution between G_3_ and C_3_ as well as A_3_ and U_3_ [30]. Thus it appears there are other factors besides nucleotide composition affecting microsporidian codon usage.

### Neutrality plot analyses

**Fig 4:**
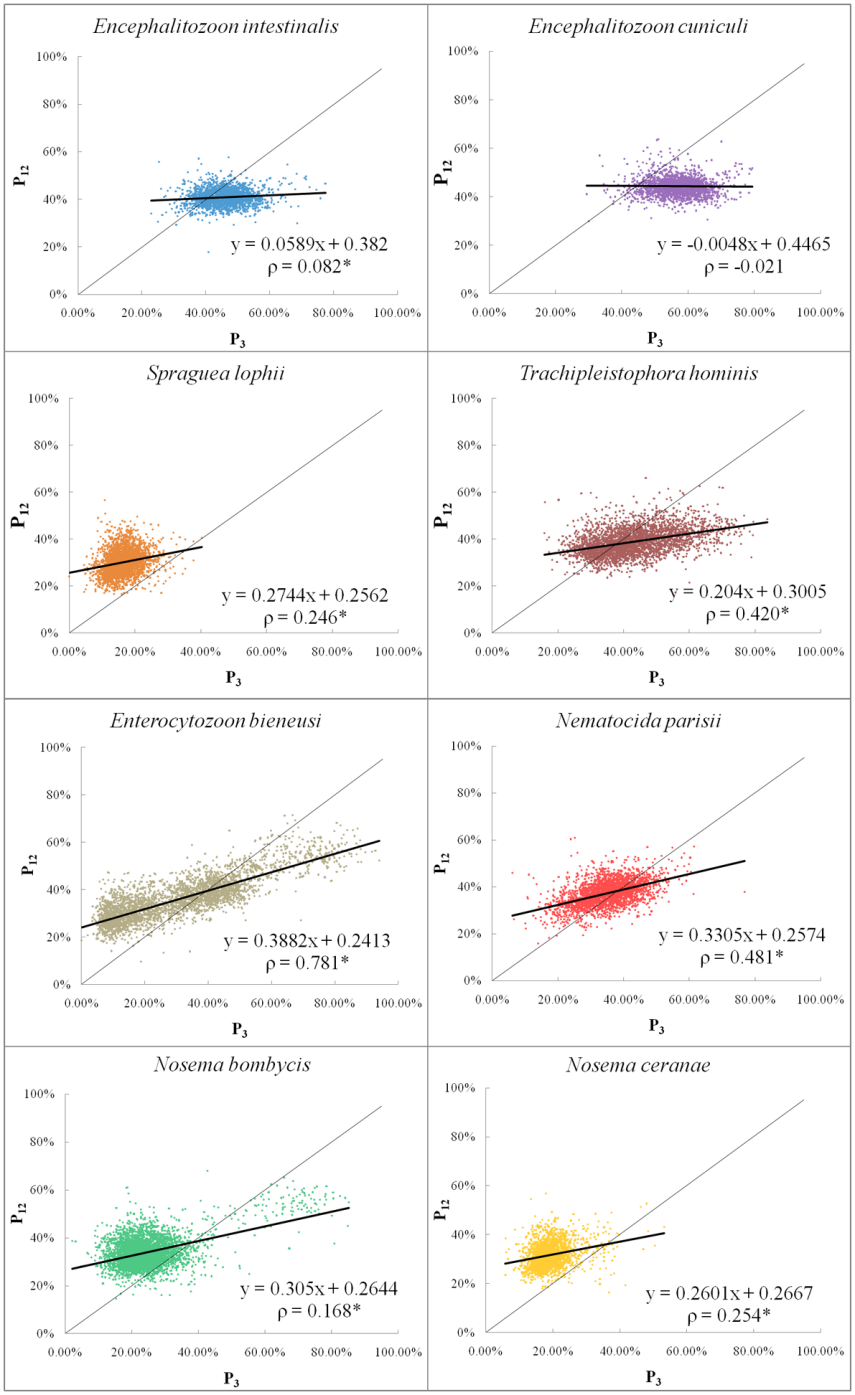
Neutrality plots of microsporidian genomes. Individual genes are plotted based on the mean GC content in the first and second codon position (P_12_) versus the GC content of the third codon position (P_3_). Regression lines and Spearman’s rank correlation coefficients (ρ) are shown, with the asterisk (*) denoting *P*-values < 0.01.

The neutrality plot analysis (Fig 4) was carried out to characterize the correlation among the three codon positions, and then identify the presence of selective mutation on CUB [25]. In the neutrality plot, if a gene is located on the slope of unity there is a significant correlation between its P_12_ and P_3_, meaning the gene is under neutral mutation via random selection pressure. If the gene is under a directed mutational pressure it should fall below the slope of unity, closer to the X-axis. Thus a regression line with a slope less than 1 would indicate a whole genome trend of non-neutral mutational pressure [31]. The *Encephalitozoon* species have regression slopes of 0.0589 and -0.0048, and correlation coefficients of 0.082 and -0.021 respectively. Their extremely low relative neutralities (5.9% and 4.8%) might suggest a large amount of directed mutational pressure, although the low Spearman correlations combined with *P*-values > 0.05 make these unreliable (Fig 4 and Table 4). *S*. *lophii*, *T*. *hominis*, *E*. *bieneusi*, *N*. *parisii*, *N*. *bombycis* and *N*. *ceranae* all have relative neutralities ranging from 20–38% and significant correlation coefficients with *P*-values < 0.01 (Fig 4 and Table 4), indicating that directed mutational pressure plays an important role in shaping CUB for these Microsporidia.

### Correspondence analyses

**Fig 5:**
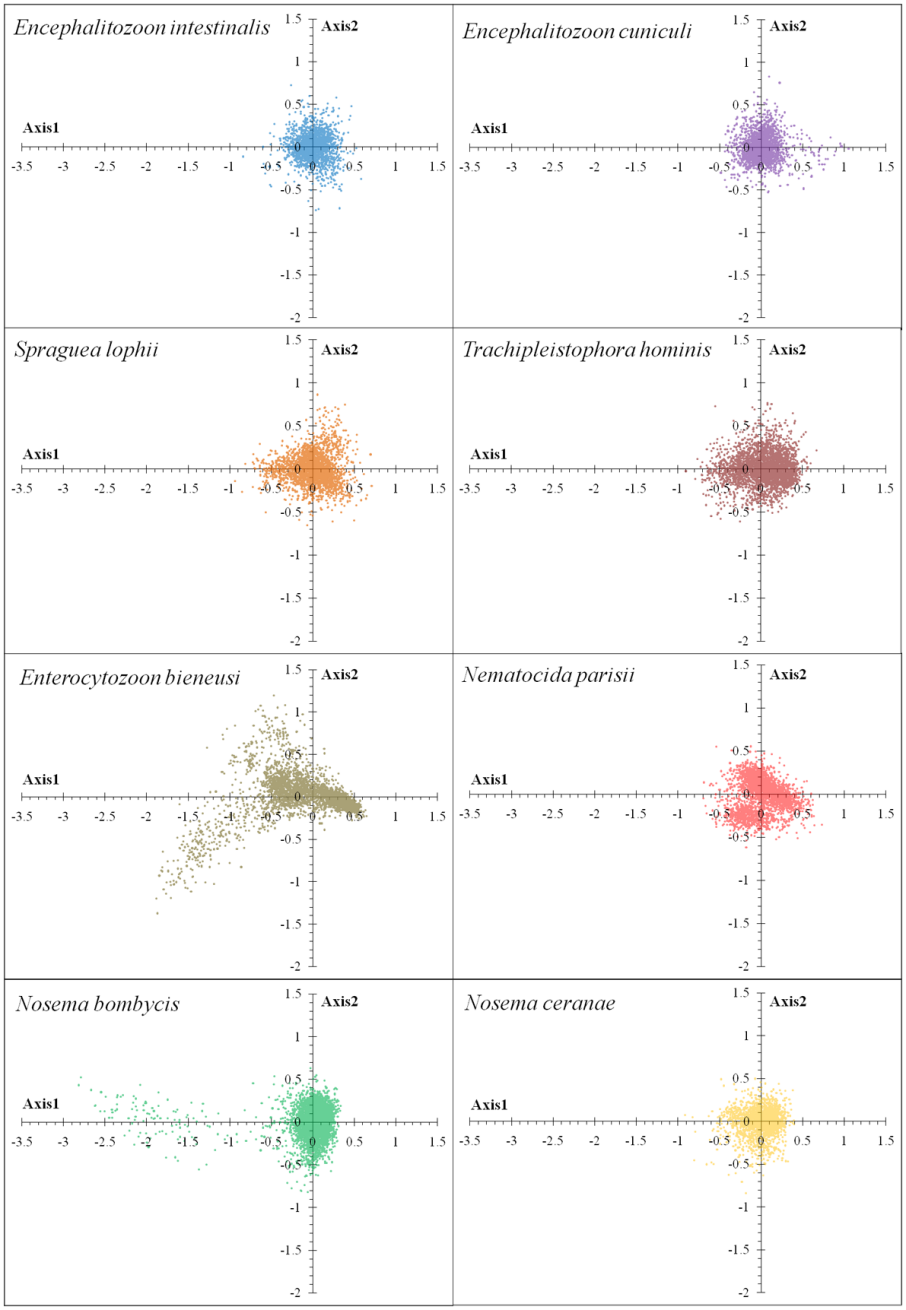
The correspondence analysis (COA) of the genes in microsporidian genomes. Each point represents a gene corresponding to the coordinates of the first and second axes of variation generated from the correspondence analysis. Some *E*. *bieneusi* and *N*. *bombycis* genes scattered to the left of Axis 1 might have distinct codon usage biases.

The correspondence analysis was used to check what other factors shape the microsporidian CUB. This multivariate statistical method surveys the variation of RSCU values within the genome [28]. The correspondence analysis shows the distribution of genes and reflects the distribution of their corresponding codons, unveiling potential influences on CUB [13]. In the correspondence analysis, a series of orthogonal axes were produced to represent the factors responsible for CUB (Fig 5). For *E*. *intestinalis*, *E*. *cuniculi*, *S*. *lophii*, *T*. *hominis*, *E*. *bieneusi*, *N*. *parisii*, *N*. *bombycis*, *N*. *ceranae*, Axis 1 accounted for 9.81%, 11.98%, 12.44%, 16.42%, 18.06%, 12.61%, 12.29%, 9.59% of their respective total variation; Axis 2 accounted for 8.81%, 8.69%, 7.33%, 6.33%, 8.32%, 10.45%, 8.98%, 6.67% of variation, respectively; and the first four axes combined (Axes 1 through 4) accounted for 26.51%, 31.01%, 31.08%, 33.29%, 35.24%, 34.92%, 31.31%, 24.54%, respectively. This suggests that the first axis is the primary factor (9–18% of the overall variation), which was also found to be significantly correlated with CAI, ENc, and GC_3s_. However, other factors are also responsible for the codon usage variation based on Axes 2 to 4.

### Codon usage indices

**Fig 6:**
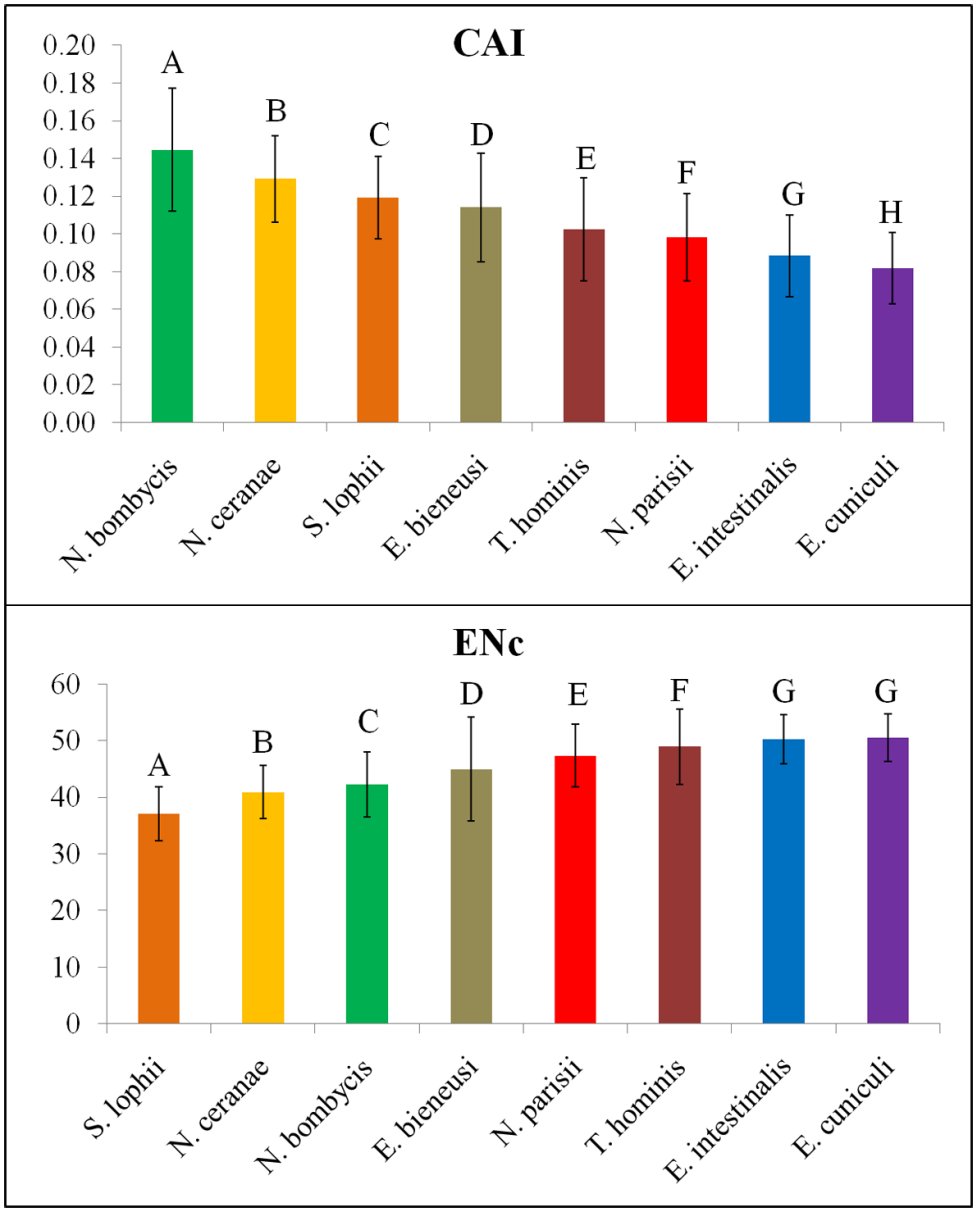
The variance analysis of codon indices (CAI and ENc) among microsporidian genomes. The one-way ANOVA shows high *F*-values (1734.906 in CAI, 1919.114 in ENc) and significant *P*-values (0.000 in both), strongly supporting the differences among microsporidian genomes.

The notable differences observed in CUB between *Encephalitozoon* species and the other microsporidians (Fig 1) were confirmed by one-way ANOVA (*F*-value: 1734.906 in CAI, 1919.114 in ENc; and *P*-value < 0.01 in both) based on the CAI and ENc (Fig 6) and by a T-test (*P*-value < 0.01). CAI is a ratio of the synonymous codon bias in a gene to a highly expressed reference gene. With values that range between 0 and 1, a higher CAI value indicates a stronger bias of synonymous codon usage and a potentially higher gene expression [32]. For microsporidian genomes, their mean CAI values are less than 0.15, and their mean ENc values are larger than 37 (Fig 6). These indicate the presence of mild synonymous codon usage bias across microsporidian genomes as a whole, with *Encephalitozoon* species showing the highest degree of randomization (Fig 6).

## Discussion

The codon usage bias, an important feature of species that can reflect the evolutionary patterns of their genome, has been reported in numerous organisms [33]. Here, the CUB in Microsporidia was studied based on eight genomes and 22,467 coding sequences, with optimal codons identified by RSCU values. The optimal codon usage pattern was found to be significantly different between species from the genus *Encephalitozoon* and those from other microsporidian lineages. Nearly all of the optimal codons in *S*. *lophii*, *T*. *hominis*, *N*. *parisii*, *E*. *bieneusi*, *N*. *bombycis*, *N*. *ceranae* feature a biased A/U third codon position, while the *Encephalitozoon* species (*E*. *intestinalis*, *E*. *cuniculi*) have a more balanced nucleotide distribution, yet slightly biased towards G/C. Although the microsporidian CUB are mild according to CAI and ENc values, these statistics are likely the result of a larger distribution of varying biases for individual genes averaging out to a less significant overall genome bias. This has previously been described [3, 5], where *Encephalitozoon* GC content smoothly arced across the chromosomes, but averaged an unremarkable GC%. Still, nucleotide composition is clearly a factor in microsporidian CUB, which was confirmed by correlation analysis.

Besides nucleotide composition, the microsporidian CUB is also influenced by other factors including directional mutation pressure, which appears to play a much larger role than selection in the CUB of Microsporidia according to neutrality analyses. While the neutrality plots do suggest an even larger directional mutation pressure for the *Encephalitozoon* species, the observed correlation coefficients undermine its overall significance. Being the first completely sequenced microsporidian genome, *Encephalitozoon cuniculi* has long been regarded as the model Microsporidia. Its reduction and compaction were for a time thought to be typical traits of microsporidian genomes. However, comparative analyses of recently released microsporidian genomes [6] rather indicates that *Encephalitozoons* are the exception rather than the norm, and that the evolutionary trends they have followed are not characteristic of the group. This is corroborated by our analyses. As the *Encephalitozoon* species are known as the smallest eukaryotic genomes (assembled genomic sizes of 2.22 and 2.50 Mbp; Table 3), they are likely under stronger reductionist pressure (directional mutation pressure) than the larger microsporidian genomes, of which many have expanded by genome duplication, horizontal gene transfer and transposable elements proliferation [1].

Intuitively, different hosts respond differently to parasitic infections, and while arthropods do possess an innate immunity, they lack the adaptive immune response found in mammals. However, the *Encephalitozoon* species represented here are not the only microsporidians infecting mammals, with both *E*. *bieneusi* and *T*. *hominis* reported as human pathogens [18, 22]. If host specificity was the sole factor involved, we would expect these four species to display similar trends, which is clearly not the case. Unfortunately, it is unknown how long these species have been coevolving with their host, and the observed disparities may be due to differences in duration rather than in the relative strengths of the underlying evolutionary pressure applied. Here, we hypothesize that the *Encephalitozoon* species may have been under mammalian host pressure for longer evolutionary periods, which would explain why they are so markedly different. A caveat of this is that the host specificity of these organisms is unclear. In fact, the *Encephalitozoon* species *E*. *romaleae* infects grasshoppers but also most likely can pester mammalian cells based on its uncanny genomic similarities with is sister species *E*. *hellem* [4], and we don’t know if its presumed absence in humans is real or rather due to limited sampling. Alternatively, the observed differences may be a direct consequence of the strongly reduced metabolic potential inherent to their Lilliputian gene repertoire, and it would be interesting to revisit the genome of the closest known relative of *Encephalitozoon* species, *Ordospora colligata* [6], which has been released during the latter stages of publication of this manuscript. *O*. *colligata* infects the water flea *Daphnia* (an arthropod [34]) and displays a similarly reduced genome, whose overall characteristics may help better delineate what has happened in the lineage leading to the *Encephalitozoon* species.
